# Review of microstructure and properties of low temperature lead-free solder in electronic packaging

**DOI:** 10.1080/14686996.2020.1824255

**Published:** 2020-10-19

**Authors:** Kai-Kai Xu, Liang Zhang, Li-Li Gao, Nan Jiang, Lei Zhang, Su-Juan Zhong

**Affiliations:** a School of Mechatronic Engineering, Jiangsu Normal University, Xuzhou, Jiangsu, China; b State Key Laboratory of Advanced Welding and Joining, Harbin Institute of Technology, Harbin, Heilongjiang, China; c Shanghai Institute of Radio Equipment, Shanghai Institute, Shanghai, China; d State Key Laboratory of Advanced Brazing Filler Metals and Technology, Zhengzhou Research Institute of Mechanical Engineering, Zhengzhou, China

**Keywords:** Low temperature solder, wettability, microstructure, mechanical properties, oxidation resistance, 103 Composites, 308 Materials resources / recycling, 503 TEM, STEM, SEM

## Abstract

Low temperature solder (In-based, Sn-Bi, Sn-Zn) has great advantages in aerospace and through-hole technology assemblies in IBM mainframe due to its unique low temperature characteristics. The review evaluates the effects of alloying elements, rare earth elements and nanoparticles on the wettability, microstructure, mechanical properties and oxidation resistance of the low-temperature solders.

## Graphical abstract

**Figure:**
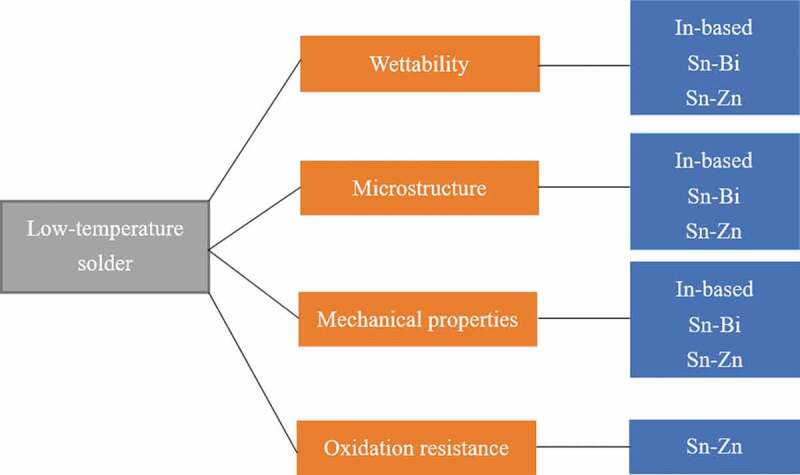

## 1. Introduction

For a long time, Sn-Pb solder has been widely used in the field of electronic packaging because of its excellent characteristics, such as low melting point (183 °C), good wettability and low price [1,2]. However, Sn-Pb solder is toxic. The use of lead has been greatly reduced with the increasing awareness of environmental protection and the issuance of ‘lead restriction order’ by many countries [3,4]. The researchers have to study new lead-free solder. In addition, with the development of electronic components to the direction of micro element, the requirements of solder joints in electronic packaging components are higher and higher, which makes the size of solder joints smaller and smaller [5].

At present, the new type of lead-free solder mainly includes Sn-Cu, Sn-Ag, Sn-Bi binary solder and Sn-Ag-Cu ternary alloy solder. The melting point of Sn-Ag solder is high (221 °C), and the price of Sn-Ag lead-free solder is relatively high due to the presence of Ag, which limits the use of the solder [6]. The cost of Sn-Cu lead-free solder is low, and the raw materials are wide. The melting point of Sn-Cu solder is high (227 °C), which limits the use of the solder [7]. Sn-Ag-Cu solder has good solderability, wettability and creep resistance. It is considered as one of the ideal substitutes for Sn-Pb solder [8]. However, there are some disputes about the element proportion of Sn-Ag-Cu composite solder. At present, the main problem of the new composite solder is that the melting point temperature is high, but the melting point of the low-temperature alloy solder is low, and the low-temperature lead-free solder has good fatigue resistance and ductility, which has good wettability for glass and ceramics.

The review evaluates the effects of alloy elements (Ag, Cu, Zn etc.), nanoparticles (CNTs, GNSs etc.) and rare earth elements (La, Nd, Pr etc.) on the wettability, mechanical properties, microstructure and oxidation resistance of low-temperature lead-free solder (In-*X*, Sn-Bi, Sn-Zn), and points out the potential problems and future research directions of low temperature lead-free solder.

## 2. Wettability

### 2.1. In-based low temperature lead-free solder

In-based solder has a very low melting point, and has good conductivity, thermal conductivity, fatigue resistance and ductility. It is a very good aerospace material [9]. In-based solder can form good metallurgical connection with ceramics, glass and low temperature materials. It is a good heat conduction interface material and has high application value [10].

**Figure 1.**
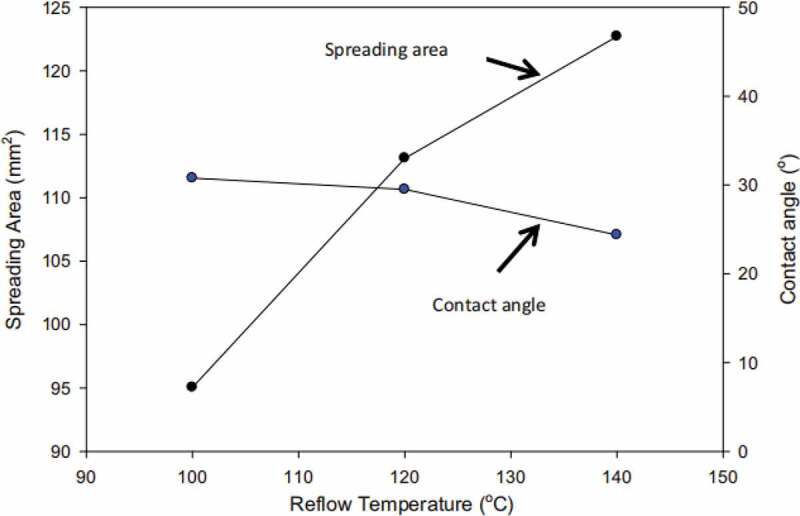
Spreading area and contact angle of In-Bi-Zn solder using different temperature on Cu substrate. Reprinted with permission from Ervina Efzan Mhd Noor et al. [15]. Copyright 2015 Springer nature.

Gan et al. [11] prepared In-7Ag alloy solder by induction melting. The microstructure and properties of the alloy solder were studied. They found that the addition of Ag element improved the wettability of In-Ag solder, and the melting point of In-Ag solder can be changed by adjusting the amount of Ag addition to meet different soldering requirements. It can adjust melting point and improve wettability at the same time due to Ag element has high melting point (961 °C) and good wettability. As a low temperature soldering material, In-Ag solder must flow into the interface between the soldering seam and the base metal under the specified soldering temperature. When the liquid solder enters the soldering seam, the cast soldering seam will be solidified [12,13]. In-Ag is a low temperature lead-free solder alloy. Prasad et al. [14] studied the relationship between the wettability of In-Sn-Zn ternary alloy solder and the content of Sn and Zn. The results showed that the surface tension of the composite solder was increased with the addition of two elements, and the wettability of the solder got worse. In the process of soldering, there are two systems (In-Zn, Sn-Zn) in the in Sn-Zn solder. The segregation of Sn atom and the formation of similar diatomic clusters make the wettability of the liquid solder decrease. Because of the activity of In and the oxidizability of Zn, the reaction sequence of the two systems is difficult to grasp. The microstructure and properties of In-32.7Bi-0.5Zn composite solder after repeated reflowing was investigated by Noor et al. [15]. The results are shown in Figure 1. DSC shows that the solder In-32.7Bi-0.5Zn alloy has a lower melting temperature (72.3 °C). The lowest melting temperature ensures that the solder melts successfully, which forms a connection with the substrate, and redissolve in the shortest possible process time. As the reflux temperature increases from 100 °C to 140 °C, the spreading area increases and the contact angle decreases. With the increase of temperature, the surface tension between the molten solder and the substrate or between the liquid solder and the flux is reduced, the flow speed of the liquid solder is increased, and the wettability of the solder is improved.

### 2.2. Sn-Bi low temperature lead-free solder

**Figure 2.**
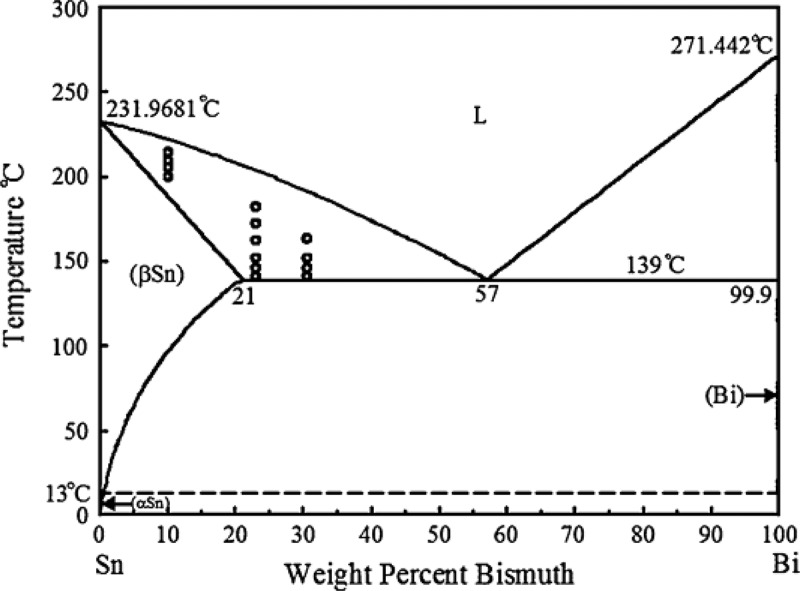
The phase diagram of Sn-Bi binary system. Reprinted with permission from C.- H Yeh, et al. [16]. Copyright 2010 Springer nature.

Figure 2 is a typical Sn-Bi phase diagram [16]. It can be seen from the diagram that the eutectic composition of the solder is Sn-58Bi, the eutectic melting temperature is 138 °C, and the eutectic temperature is close to the traditional Sn-Pb alloy solder. Sn-Bi solder has very good processability. Low melting point solder has great advantages in the field of electronic packaging, whether it is three-stage or above electronic packaging, or three-stage or below electronic packaging which is more sensitive to temperature. In the process of soldering, the materials with different coefficient of thermal expansion have great advantages because of the mismatch or even damage of components due to high temperature. A more uniform bonding area was achieved when the Sn-Bi solder was used. It can reduce the generation of bubbles, and meet the requirements of low temperature air tightness [17].

Wettability refers to the spreading ability of molten solder on the surface of base metal [18]. In the process of soldering, the liquid solder will be wetted, flowed and spread on the surface of the base metal, and then the whole weld will be filled with the help of capillary action. Wetting is an important prerequisite for the formation of solder joints. Therefore, wettability is one of the important criteria to evaluate the quality of solder. The traditional Sn-Pb solder has good wettability because of the existence of Pb. Some researchers have made a series of research to improve the wettability of Sn-Bi composite solder. The alloy characteristic of Sn-Bi solder is that Bi is solid soluble in Sn matrix and does not form metal compound [19]. The effect of Bi addition on the wettability of Sn-Bi lead-free solder on Cu substrate was studied by Chang et al. [20], and the results are shown in Table 1. It is found that the spreading area of Sn-Bi solder increases gradually with the increase of Bi content. This is mainly due to the fact that Bi is a surface-active element. The increase of Bi reduces the solid-liquid interfacial tension between the molten alloy solder and the Cu substrate, and enhances the wetting force of the molten solder on the Cu substrate. He et al. [21] studied the effect of adding Ag on the wettability of Sn-Bi solder on Cu substrate. The results showed that adding appropriate Ag element can improve the wettability of Sn-Bi solder. The Ag element has good oxidation resistance, which reduces the surface tension between the liquid solder and the flux. At the same time, the generation of Ag_3_Sn grains will hinder the reaction between Sn and Cu substrate, and improve the wettability of Sn-Bi solder. The relationship between Cu content and the wettability of Sn-Bi solder on Cu substrate was studied by Zang et al. [22]. They point out that the interfacial tension between the molten solder and the flux is reduced due to the oxidation resistance of Cu element when 0.5 wt. % Cu was added into the solder. Therefore, the wettability of Sn-Bi solder is improved after adding proper amount of Cu. Chen et al. [23] found that the wettability of Sn-Bi solder is inversely related to the content of Zn, which is attributed to the oxidation of Zn. Zn element is easy to oxidize. The fluidity of molten solder was reduced because Zn element can oxidize the surface of Sn-Bi composite solder, which reduced the wettability of solder. Mokhtari et al. [24] also obtained similar results through experimental research. Wang et al. [25]studied the relationship between Al element and the wettability of Sn-Bi solder. He found that the wettability and spreading area of Sn-Bi solder decreased gradually with the increase of Al content. Al element oxidized to form a compact oxide film in the process of soldering, which covers the surface of molten solder. It hinders the flow of liquid solder and makes the wetting effect of solder worse. Chen et al. [26] found that with the addition of In, the wetting area of Sn-Bi solder increased first and then decreased. The flow rate of the liquid solder is increased and the wettability is improved when a small amount of In is added. However, the flow of solder was hindered due to the excessive oxidation of In when excessive In was added, which deteriorates the wettability of Sn-Bi solder. Zhang et al. [27] also studied the relationship between the content of Sb element and the wettability of Sn-Bi solder. The results showed that when the content of Sb was more than 0.5 wt. %, the wettability of Sn-Bi solder began to decrease. The wetting ratio of Sn-Bi-Sb solder is mainly affected by three factors: liquidus temperature, physical wetting and reactive wetting [28,29]. In this experiment, the wettability of solder is mainly affected from two aspects: a) Bi element can improve the degree of overheating and wettability of solder; Too much Bi (too low Sn) results in a decrease in reactive wetting. b) In the range of 0 and 0.5 wt. %, the higher the Sb, the faster the reactive wetting, but the lower the superheating. There were too many high melting point Cu-Sb compounds on the surface of the base metal when the content of Sb was too high. Cu-Sb compounds accumulated on the wetted base metal surface, which hindered the further wetting of the base metal and reduced the wetting area. In order to improve the properties of Sn-58Bi lead-free solder, 0–0.4 wt% CuZnAl particles were added into the low-temperature eutectic Sn-58Bi lead-free solder by Yang et al. [30]. The results show that the spreading area reached the maximum when the addition of CuZnAl was 0.2 wt. %.

**Table 1.** Spreading area of Sn-*x*Bi alloy solder.

Sn-*x*Bi	Sn-5Bi	Sn-10Bi	Sn-15Bi
Spreading area(mm^2^)	49.6	57.6	63.4

As we all know, rare earth elements (La, Pr, Nd, etc.) are called ‘vitamins’ of metal elements, A small amount of rare earth elements can change the properties of solder [31]. As a large country of rare earth, China is rich in rare earth. The study of rare earth elements has a profound impact on the properties of composite solder. Dong et al. [32] studied the wettability of Sn-58Bi composite solder by adding rare earth element Ce. The results showed that the melting point of the solder did not change significantly, but the wettability area of the composite solder reached the maximum when the content of Ce was 0.1 wt%. This is mainly because Ce is a surface active element with a large atomic radius. Ce element does not dissolve in the solder. It is usually dispersed on the solder surface, which reduces the surface tension between the solder and the flux, and makes the solder fully wetted in the base metal. Liu et al. [33] reported that adding 0.5 wt.% Ni nanoparticles to Sn-Bi-Ag solder can effectively improve the wettability of the composite solder. Nano Ni elements gather on the surface of the liquid solder to reduce the surface tension of the solder due to the strong surface activity of Ni nanoparticles, thus enhancing the fluidity of the liquid solder. Gain et al. [34] also found that the wetting area of Sn-Bi solder on Cu substrate was about 20% higher than that of Sn-Bi solder when Y_2_O_3_ nanoparticles were added to Sn-Bi solder.

### 2.3. Sn-Zn low temperature lead-free solder

**Figure 3.**
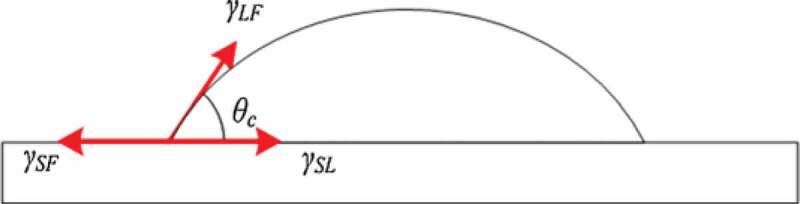
Schematic diagram of the surface tension under equilibrium state.

Sn-Zn lead-free solder has a wide range of raw materials and a low melting point, which is close to the traditional Sn-Pb solder. It is one of the options to replace Sn-Pb solder at present. However, the use of Sn-Zn solder is limited due to its coarse structure, poor wettability and oxidation resistance [35]. The effect of adding noble metal Ag on the wettability of Sn-Zn solder was studied by Wu et al. [36]. The experimental results are shown in Table 2. It can be seen from the table that the wettability of the alloy solder is improved by adding appropriate amount of Ag element. The addition of trace Ag element can improve the oxidation resistance of Sn-Zn solder. Ag element can react with Zn to form Ag-Zn compound, which reduces the generation of ZnO oxide, and increase the wetting area. Haitao et al. [37] reported that adding Bi element can improve the wettability of Sn-Zn solder. Bi element is easy to gather on the surface, and its melting point is relatively low. Bi is conducive to diffusion, which can reduce the surface tension of liquid solder and improve the wettability of solder. Daly et al. [38] Showed similar results through experiments. Yu et al. [39] found that the wettability of Sn-Zn alloy solder can be improved by adding proper amount of Cu. Cu atom reacts with free Zn atom to form Cu-Zn compound, which consumes excessive Zn atom. Then it can reduce Zn atom participating in oxidation reaction, which reduces surface tension of liquid solder and improves wettability of Sn-Zn solder. Huang et al. [40] reported the effect of Zn element on the wettability of Sn-Zn solder. The results show that compared with Sn-9Zn solder, Sn-6.5Zn has a low surface tension and spreading ratio, which means that Sn-6.5Zn solder has better wettability to Cu than Sn-9Zn solder. Zn affects the wettability of Sn-Zn solder and Cu substrate from the following two aspects: a) Excessive Zn can enhance the degree of overheating which favours the wetting. b) Higher Zn will impede the wetting because they cause more severe oxidation of the melt. The effects of both sides compete with each other is that result in an optimum Zn concentration for wetting is around 6.5 wt% rather than 9 wt%. The literature [41] shows that Ni and Cu have similar surface activity. Ni added into Sn-Zn solder can reduce the melting point of Sn-Zn solder and improve its wettability. Ni element gathers on the surface of solder during soldering because of the surface activity of Ni element, which decreases the surface tension of solder and improves the wettability. Wang et al. [42] Found that the wetting effect of Sn-Zn solder can be effectively improved by adding a proper amount of Al element. Al atom is easy to react with Cu and Zn to form Cu-Al compound and Cu-Zn Compound. The compounds are strongly diffused in the formation process, so that the wettability of Sn-Zn alloy solder can be improved. However, Chen et al. [43] has come to the opposite conclusion. They believed that Al element will react with oxygen to form a dense Al_2_O_3_ oxide film, which hinders the flow of liquid solder. It may be due to the different addition proportion of Al element, resulting in two different results. Du et al. [44] added P element to Sn-Zn-Ce ternary composite solder, and found that proper P element can effectively improve the wettability of the solder. Chen et al. [45] studied the relationship between the content of Ga element and the wettability of Sn-Zn solder. The results showed that adding appropriate Ga element can reduce the wettability angle of Sn-Zn solder and increased the wettability spreading area. The wetting angle is determined by Young-formula [46], the schematic diagram of the surface tension under equilibrium state is shown in Figure 3.

(1) $$cos\theta =\frac{\left(\gamma_{SF}-\gamma_{SL}\right)}{\gamma_{LF}}$$

**Table 2.** Testing results of solder wettability.

Experiment Number	Solder type	Spreading area (mm^2^)	Wetted area ratio with Sn-37Pb solder
1	Sn-37Pb	185	1
2	Sn-9Zn	83	0.49
3	Sn-9Zn-1Ag	101	0.55

Where θ represents wetting angle, $\gamma_{SF}$ represents solid-gas surface tension, $\gamma_{SL}$ represents liquid-solid surface tension, $\gamma_{LF}$ represents gas-liquid surface tension.

**Figure 4.**
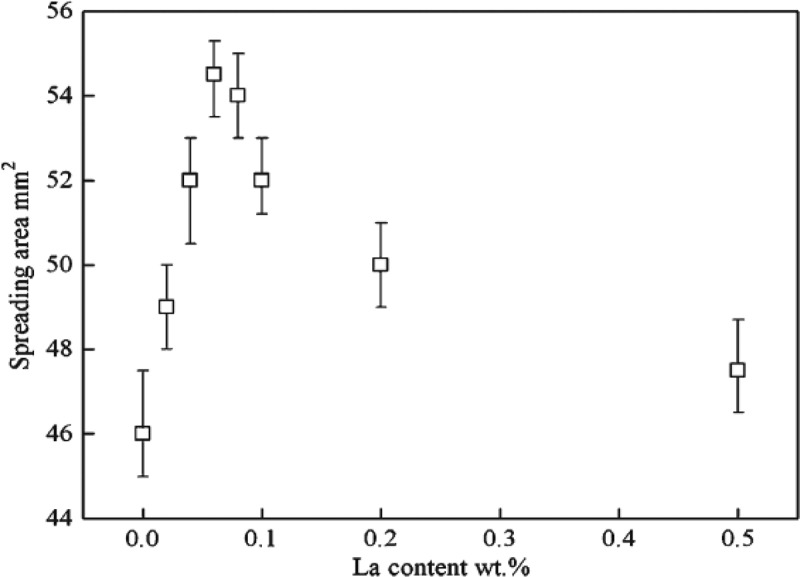
Spreading area of Sn9Zn-*x*La solder joints.

Zhang et al. [47] studied the influence of rare earth (RE) on the wettability of Sn-Zn lead-free solder. The results are shown in Figure 4. It can be found that the wetting area reaches the maximum when La content is 0.08 wt%. This is because the RE element has ‘Sn affinity’. It is easy to react with Sn to form rare earth phase particles when the amount of RE element is small. The particle size is small, it is easy to change the liquid-solid-gas three-phase equilibrium of the liquid solder and reduce the surface tension of the liquid solder. Rare earth phase particles increase when La was added too much, which is easy to react with oxygen to form large oxide slag and reduce the wettability of solder. Hu et al. [48] found that adding trace rare earth Nd element to Sn-Zn solder can improve the wettability of the solder. Nd is a surface-active element. It can reduce the surface tension of molten solder and improve the fluidity of liquid solder at low temperature. However, the oxidation of Nd is serious with the increase of temperature when the amount of Nd is too much, which hinders the further spreading of solder and reduces the wettability of Sn-Zn composite solder. Zn atom reacts with Cu substrate to form Cu-Zn compound in Sn-Zn solder/Cu substrate, which hinders the diffusion of Sn atom to Cu substrate. This phenomenon is not found in other Sn-based solder. This can reduce the wettability of Sn-Zn-Ga solder. The reactive wetting between the solder and Cu substrate is promoted after rare earth Pr element is added to the solder, and the wetting area is increased [49].

Table 3 summarizes the effect of trace element addition on the wettability of In-based/Sn-Bi/Sn-Zn solder. It can be seen from Table 3 that some metal materials, rare earth and nanoparticles can improve the wettability of the low temperature solders (In-based/Sn-Bi/Sn-Zn) to a certain extent, which is conducive to improving the reliability of solder joints. The wettability of solder is an important criterion to evaluate the reliability of solder joint, because good wettability of solder can ensure well interconnection between molten solder and base metal.

**Table 3.** Effect of trace element addition on the wettability of In-based/Sn-Bi/Sn-Zn solder.

Solder	Elements	wt.%	Spreading area	Contact angle	wetting time	reliability	Reference
In	Ag	7	↑				[11]
	Sn, Zn	20, 10	↑				[14]
	Bi, Zn	32.7, 0.5	↑↑	↓			[15]
	Bi	15	↑ (12%)				[20]
	Ag	3.7	↑				[21]
	Cu	0.5		↓↓ (52%)		√	[22]
	Zn, Al	2, 0.05	↑				[25]
Sn-Bi	In	2.5	↑ (15%)				[26]
	Sb	2	↑ (21.2%)			√	[27]
	Ce	0.1	↑↑				[32]
	Nano-Ni	0.5	↑				[33]
	Ti nanoparticle	0.1	↑	↓			[114]
	Ag	1	↑			√	[36]
	Bi, Ni	3, 1		↓↓			[37,38]
	Cu	10		↓↓ (55%)			[39]
	Zn	6.5	↑↑ (31.2%)				[40]
	Ni	0.1		↓			[41]
Sn-Zn	Al	0.005–0.02	↑↑				[42]
	Al	0.6		↑			[43]
	Ag, Ga	0.5, 0.5	↑				[45]
	RE(La)	0.08	↑ (18%)				[47]
	Nd	0.06	↑↑ (33.3%)			√√	[48]
	Pr	0.08			↓↓ (50%)	√	[49]

Notes:↓ = slightly decreased ↓↓ = remarkably decreased ↑ = slightly increased ↑↑ = remarkably increased √ = slightly improved √√ = remarkably improved

## 3. Microstructure

### 3.1. In-based low temperature lead-free solder

**Figure 5.**
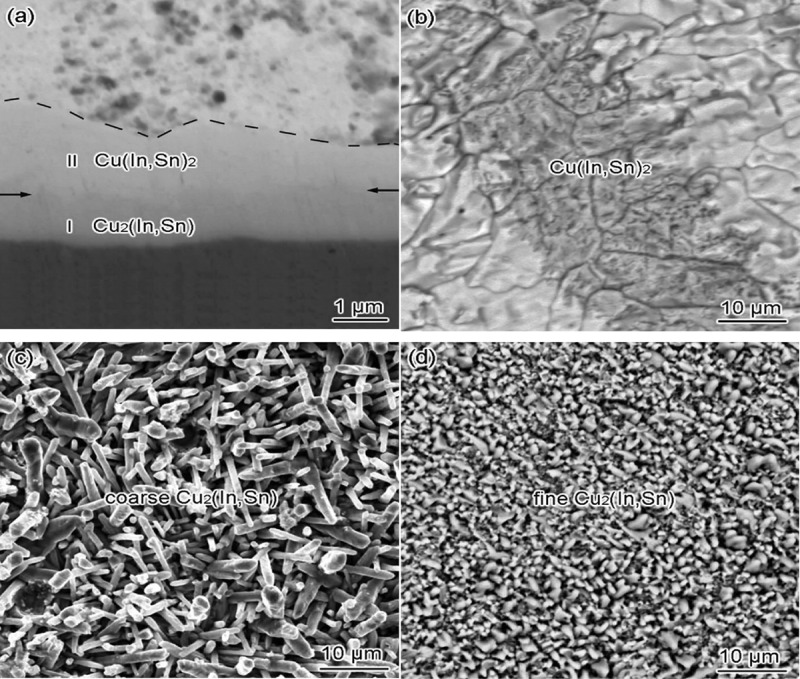
(a) Cross-sectional SEM image of the interface after soldering, (b) the top-view SEM images of reflowed chunk-type Cu (In, Sn)_2_, (c) coarse-grain Cu_2_(In, Sn), (d) fine grain Cu_2_(In, Sn). Reprinted with permission from Feifei Tian, et al. [51]. Copyright 2018 Elsevier.

Joanna et al. [50] investigated the growth of Cu/In-22Bi/Cu intermetallic compound. They found that there are two forms of intermetallic phase (Cu_11_In_9_) when the soldering temperature is 85°C-200°C. The first form is a continuous layer formed on the Cu substrate, which contains 6 wt.% Bi elements. The second form is scallop like compound layer. The compound layer is penetrated by molten liquid solder. It is the main channel of atom diffusion. The Bi-rich Cu_11_In_9_ phase becomes CuIn_2_ phase with the increase of temperature. The diffusion channel is blocked due to the growth of IMC. The growth rate of intermetallics decreases, and the interface microstructure becomes uniform. Tian et al. [51] studied the interface reaction between In-48Sn solder and polycrystalline Cu substrate in the process of solid-state aging. Figure 5 shows a scanning electron microscopy (SEM) image of the microstructure after refluxing. It is found that two IMCs are formed in three sublayers from solder to substrate side after reflow at 160 °C for 5s. They are Cu (In, Sn)_2_ layers with tetragonal crystal structure, Cu_2_(In, Sn) sublayers with coarse grains and Cu_2_(In, Sn) sublayers with fine grains. The shape of Cu (In, Sn)_2_ grain is the block with the largest grain, the shape of Cu_2_(In, Sn) grain is rod with medium grain size, and the shape of Cu_2_(In, Sn) grain with the smallest grain size is shown. Cu (In, Sn)_2_ layers grow and consume Cu_2_(In, Sn) layers continuously because the diffusion rate of In and Sn atoms is higher than that of Cu atoms after aging at 40 °C. Jin et al. [52] studied the relationship between the microstructure of In-Bi solder and its composition ratio. They found that Bi_3_In_5_ was only detected in In-50Bi alloy solder, BiIn_2_ was detected in In-40Bi, In-33.7Bi and In-30Bi, which confirmed that In-Bi binary system contained stable mesophase: Bi_3_In_5_, BiIn_2_ and ɛ.

### 3.2. Sn-Bi low temperature lead-free solder

The molten solder reacts with the base metal to forms the solder joint after cooling in the process of soldering. Whether the internal structure of the solder joint is uniform or there are too many intermetallic compound layers will directly affect the service life of the solder joint. The research on the internal microstructure of the solder joint is the basis of the research on the reliability of the solder joint [53]. It is easy to accelerate the growth of intermetallic compounds in the process of Sn-Bi soldering due to the unique characteristics of Bi.

**Figure 6.**
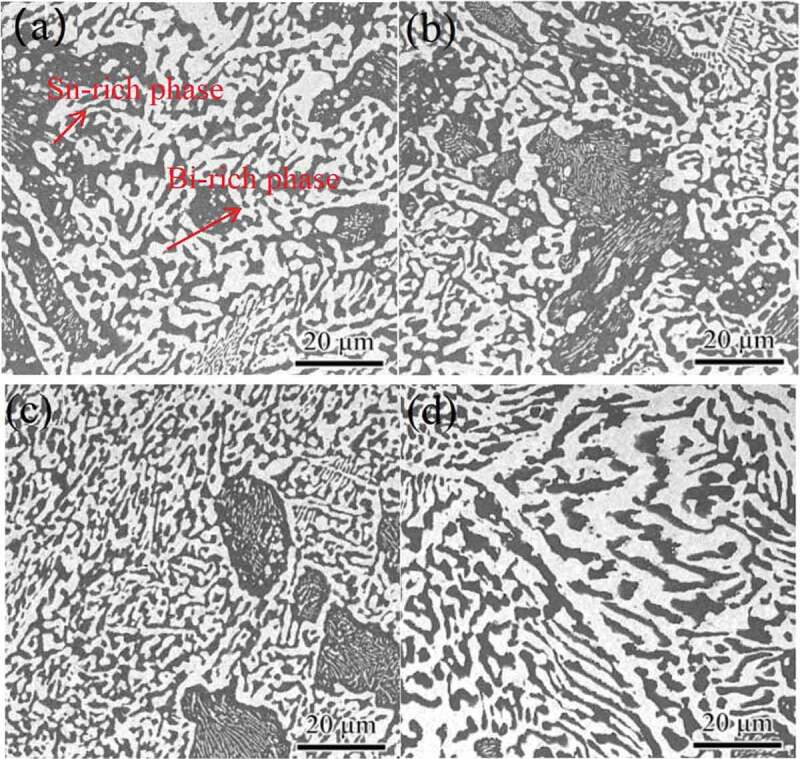
Microstructure of Sn-58Bi-*x*Sb (x = 0, 1.0, 1.5, 2.0) alloys with different Sb contents (wt.%). (a) Sn-58Bi; (b) Sn-58Bi-1Sb; (c) Sn-58Bi-1.5Sb; (d) Sn-58Bi-2Sb.

Wang et al. [54] added phosphorus to Sn-Bi solder. They found that the alloy strength of the composite solder was improved when the P content was 1 wt %. It was mainly due to the refinement and dispersion strengthening of the internal microstructure of the solder. It can decrease the crystal size of β – Sn and improve the strength of the solder joint. In addition, the intermetallic compound can refine the grain size and improve the plasticity of the composite solder. The relationship between the thickness of Sn-Bi solder/Cu intermetallic compound and Cu element was studied by Lai et al. [55]. The results showed that with the increase of Cu element, the thickness of IMC between Sn-Bi solder and Cu substrate increased gradually. The thickness of IMC increased with the increase of aging time. The diffusion amount of Cu_6_Sn_5_ compound in the liquid solder decreased due to the increase of Cu element content, and the thickness of IMC thickened. Miao et al. [56] obtained similar results through experiments. Chen et al. [57] studied the influence of In element on the structure and properties of Sn-Bi-based lead-free solder. The results showed that 42Sn-Bi solder has typical layered structure, and the eutectic structure is composed of alternate layered white Bi phase and black Sn phase. With the addition of In elements, the number of primary Sn-rich phases increases. The granular Bi phase was formed after 4 wt.% Bi was added. Bi and In particles are mainly distributed in Sn matrix and along Bi phase. It was found by Zhu et al. [58] that adding Zn element to Sn-Bi solder will preferentially produce Cu-Zn compound between Sn-Bi solder and Cu substrate, and gradually produce Cu-Sn compound with the increase of soldering time because the free energy of Cu-Zn compound is lower than that of Cu-Sn compound. The effect of Ni on the growth of intermetallic compounds in Sn-Bi solder/Cu was studied by Yang et al. [59]. The results show that Ni can inhibit the growth of Sn-Bi/Cu intermetallic compound. The particle size of Sn-Ni compound is small, which will hinder the growth of IMC in the aging process. Zou et al. [60] found that adding Al element can effectively refine the microstructure of Sn-Bi solder and improve the ductility of solder, but there is a phenomenon of coarse structure and segregation. Chen et al. [61] investigated the effect of different temperature and strain rate on the microstructure of Sn-58Bi solder. They found that the microstructure of Sn-58Bi solder consists of alternate layered two-phase (white ash area) and Sn phase (deep ash area). It has typical layered eutectic structure. A large number of nano Bi particles began to precipitate in Sn matrix with the increase of temperature and strain rate. The effect of Sb element on the microstructure of Sn-58Bi was investigated by Li et al. [62]. The test results are shown in Figure 6. It is found from the figures that coarse crystallization in Sn-58Bi solder can be eliminated to refine the structure when the amount of Sb was 1.5 wt. %. The literature [63] shows that the addition of Ag element in the aging process of Sn-Bi solder slows down the coarsening phenomenon of Bi-rich phase and inhibits the growth rate of IMC between Sn-Bi solder and Cu substrate. Zhu et al. [64] found that the addition of Cr inhibited the grain coarsening of Sn-Bi solder and refined the grain. Cr element plays a role of heterogeneous nucleation center in solder [65]. More heterogeneous nucleation particles are introduced into the composite solder to promote the refinement of solder structure. Xiong et al. [66] studied the effect of CuZnAl particles on the microstructure of Cu/Sn58Bi/Cu TLP solder joints. The results showed that CuZnAl particles in Sn-58Bi solder reduced the diffusion flux of Cu atoms. It inhibited the growth of IMCs at the interface, and significantly reduced the formation of cavities. Zhang et al. [67] also believed that CuZnAl memory particles can decrease the diffusion coefficient of IMC, which inhabits the growth of IMC at Sn-58Bi/Cu interface.

The microstructure can also be refined by adding rare earth elements. Shiue et al. [68] studied the effect of the addition amount rare earth (RE) on the microstructure through comparative experiments. They found that the microstructure was refined when the addition amount of La was 0.5 wt %. They also found that RE can not only refine the microstructure, but also inhibit the growth of IMC during aging. Chuang et al. [69] studied the effect of rare earth elements Ce on the microstructure of Sn-58Bi solder alloys. The results showed that the microstructure of Sn-Bi-*x*Ce were refined. The rare earth elements belong to the surface-active elements. According to the adsorption theory of the surface-active elements [70], the equation is as follows:

(2) $$\sum_{K}\gamma_{c}^{K}A_{K}=\sum_{K}\left(\gamma_{0}^{K}-RT\int_{0}^{c}\frac{\Gamma^{K}}{c}dc\right)A_{K}\to \min$$

Where $\gamma_{c}^{K}$ represents the surface tension of the crystal plane with adsorption of RE, $A_{K}$ represents the area of the crystal plane, $\gamma_{0}^{K}$ represents the surface tension of the crystal plane with adsorption of the RE, R represents the gas constant, T represents the thermodynamic temperature, c represents the concentration of the RE, $\Gamma^{K}$ represents the adsorption of RE at crystal plane. It can be found that rare earth elements will gather near the grain boundary or IMC, and prevent the growth of the grains or IMC by decreasing the surface energy of the grains or IMC. At the same time, the acicular Cu_6_Sn_5_ compounds in the microstructure of Sn-Bi-*x*Ce solder will disappear, which will refine the microstructure. Xia et al. [71] also reported that the size of β-Sn phase in Sn-Bi-Ag-*x*RE composite solder decreased from 20.13 μm to 8.13 μm after RE were added, so as to refine the microstructure.

Sun et al. [72] studied the effect of nano Ag particles on the microstructure of Sn-Bi solder. The results show that nano Ag particles can refine the microstructure of Sn-Bi solder. The dispersed nano Sn_3_Ag particles will be formed after the addition of nano Ag particles. They make the grains more difficult to grow, so as to refine the Bi-rich and Sn-rich phases. Liu et al. [73] pointed out that Cu_6_Sn_5_ nanoparticles can refine the microstructure of Sn-Bi solder. The melting point of Cu_6_Sn_5_ nanoparticles is high. It acts as a nucleation particle in the Sn-Bi solder after Cu_6_Sn_5_ nanoparticles was added in Sn-Bi solder. The nanoparticles can inhibit the coarsening of the Bi-rich phase. It has been pointed out in the literature [74] that after the carbon nanotubes (CNTs) were added in Sn-Bi solder, CNTs are segregated at the Sn-Bi/Cu interface during the soldering process. It hindered the diffusion of elements, which inhibited the coarsening of the interface layer and refined the microstructure of the solder. The literature [75] also shows that the thickness of IMC increases first and then decreases with the addition of Ni-coated CNTs in Sn-Bi solder, and the microstructure of solder is refined. Qiu et al. [76] reported the effect of adding Graphene nano sheet (GNSs) on the structure and properties of Sn-Bi solder. The results showed that the average grain size of Sn-Bi solder strengthened by GNSs was about 45% smaller than that of Sn-Bi solder. The micro amount of GNSs dispersed in the solder can restrain the grain growth and refine the microstructure effectively.

### 3.3. Sn-Zn low temperature lead-free solder

**Figure 7.**
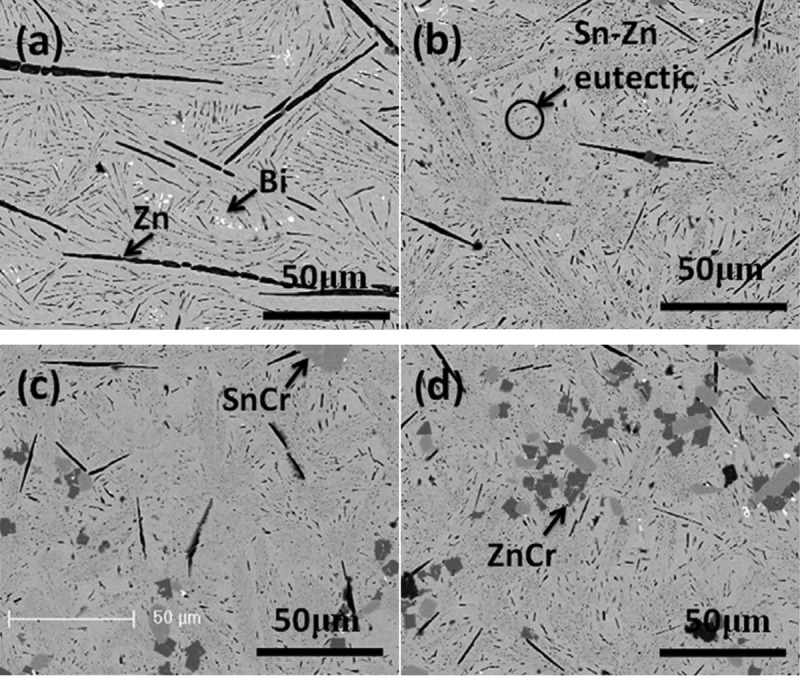
SEM micrographs of Sn-8Zn-3Bi-Cr alloys: (a) Sn-8Zn-3Bi; (b) Sn-8Zn-3Bi-0.1Cr; (c) Sn-8Zn-3Bi-0.3Cr; (d) Sn-8Zn-3Bi-0.5Cr. Reprinted with permission from Tingbi Luo, et al. [81]. Copyright 2012 Elsevier.

The effect of Ag on the microstructure of Sn-Zn solder was studied by Wu et al. [77]. They found that the microstructure of Sn-Zn solder was composed of coarse needle-like and slender rod-like Zn rich phase and broom-like Sn-9Zn eutectic structure before the addition of Ag. The matrix structure was relatively coarse. The needle-like and rod-like structures disappeared and the microstructure of solder was partially refined after Ag element was added in Sn-Zn solder. Ma et al. [78] reported the relationship between the addition of Bi element and the microstructure of Sn-Zn solder. They pointed out that the β- Sn phase of the solder matrix was refined after Bi element was added into Sn-Zn solder. The needle-like Zn phase transformation was more disordered. The trend of refinement first and then coarsening appeared with the increase of Bi content. Daly et al. [79] pointed out that the microstructure of Sn-9Zn solder will be refined when moderate Cu element was added in the solder. Cu containing alloys such as Cu_6_Sn_5_, CuZn_5_ and Cu_5_Zn_8_ are formed after Cu element was added in it. The coarse needle-like Zn-rich phase gradually becomes fine needle-like or even disappears, and the Sn-Zn eutectic structure is gradually refined. Lin et al. [80] investigated the effect of Al addition on the microstructure of eutectic Sn-9Zn solder. They found that the microstructure of eutectic solder was refined when appropriate Al was added. This is mainly due to the fact that Al belongs to the grain refiner promoting nucleation, which makes the Zn-rich phase transformation fine and the microstructure get refined. The properties of Sn-Zn solder are improved because of the addition of Bi element. On this basis, Luo et al. [81] added Cr element to study the effect on the microstructure of Sn-8Zn-3Bi-Cr composite solder. The research results are shown in Figure 7. It can be seen from the figures that a large number of needle-like Zn-rich phase and Sn-Zn eutectic phase are distributed in the microstructure of Sn-8Zn-3Bi solder. With the increase of Cr content, the primary Zn-rich phase structure gradually shortens and finally disappears, and the microstructure gradually refines. Liu et al. [82] found that the microstructure of Sn-Zn-Bi alloy solder can be refined effectively when appropriate In was added in the solder. The coarse Zn-rich phase is evenly distributed on Sn matrix in Sn-Zn solder. Cu-Sn binary phase diagram and Gibbs formation energy of Cu_5_Zn_8_ and Cn_6_Sn_5_ according to the comparison of Cu-Zn [83], Cu-Zn compound particles are generated after the addition of trace Cu element. The coarse rod structure disappears basically, so as to refine the structure. Liu et al. [84] also added the fourth element Ag to the Sn-Zn-Bi ternary solder. The results showed that the growth of IMC is inhibited after Ag was added due to the influence of diffusion mechanism. The growth direction of interface became more uniform, which formed a good metallurgical effect. Zhao et al. [85] studied the effect of rapid solidification on the properties of Sn-Zn-Bi composite solder. The results showed that Bi element dissolved in Sn matrix with dendrite structure after rapid solidification. Zn-rich phase distributed evenly in Sn matrix with 1–3 μm particles. It inhibited the growth of Sn Zn-Bi/Cu intermetallic compound, which made it more compact and uniform. Basaty et al. [86] investigated the effect of Sb element on the microstructure of Sn-Zn-Al ternary alloy solder. The results showed that appropriate Sb element can refine the microstructure of the solder. It turned the α-Zn phase in the alloy to fine needle shape, and formed uniform IMCs in the solder matrix. The effect of Ni/Sb element on the microstructure of hypoeutectic Sn-6.5Zn solder at low temperature was studied by Daly et al. [87]. The results showed that the microstructure was refined due to the dissolution effect of Sb element and the flower-like (Ni, Zn)_3_Sn_4_ intermetallic compound phase produced by Ni element. Temperature is one of the important factors that affect the metallurgical effect of solder in the process of soldering. Praphu et al. [88] investigated the effect of cooling rate on the microstructure of eutectic Sn-9Zn solder. The results showed that the needle-like Zn-rich phase gradually became thinner and shorter as the cooling rate increased from 0.03°C/s to 25 °C/s. Its distribution in the solder matrix was more uniform, and the microstructure got better refining. Liu et al. [89] studied the effect of Ti addition on the microstructure of Sn-Zn solder. It was found that the Zn-rich precipitates of the solder were obviously refined without coarse acicular precipitates. This phenomenon can be explained by the increase of the nucleation of Zn phase. The microstructure obtained is conducive to the integrity of the passive film formed on the modified surface. The influence of Na element on the microstructure of Sn-Zn solder was studied by researchers. It was found that Na can reduce the thickness of intermetallic compound layer (CuZn_4_, Cu_5_Zn_8_) formed at the interface between Sn-Zn solder and Cu substrate. Na element and Zn react to form NaZn_13_ compound according to the following reaction equation. The compounds hinder the diffusion of Zn atom, and the activation energy of Cu_5_Zn_8_ phase is higher than that of eutectic Sn-Zn phase. Too much brittle IMC will be produced if excessive Na element is added, which will affect the reliability of solder joints.

(3) $$Na+13Zn\to NaZn_{13}$$

Lin et al. [90] reported that the addition of rare earth element Ce/La can refine the microstructure of Sn-Zn solder. They pointed out that the microstructure of Sn-Zn-*x*Ce/La composite solder is composed of rod-like Zn-rich phase and eutectic. These results can be explained by the equation of nucleation rate [91]:

(4) $$P(t)=k(N/v{)}^{\frac{4}{3}}$$

Where P(t) represents the number of crystal nucleus, k represents the constant, N represents the nucleation rate, v represents the rate of crystal growth. The addition of trace rare earth element Ce/La can promote the formation of a large number of dispersed non-spontaneous nucleation. This forms fine eutectic particles and refines the microstructure of the solder. Fu et al. [92] pointed out that the microstructure of Sn-9Zn alloy can be significantly refined by adding trace rare earth element Pr. The microstructure of Sn-9Zn alloy can be significantly refined because of the strong modification of rare earth element. The coarse rod-like Zn-rich phase in the alloy disappeared, and the microstructure showed fine eutectic grains. A large number of Zn atoms are adsorbed on the surface of ZrO_2_ particles after repeated reflow when ZrO_2_ nanoparticles were added into Sn-Zn solder. It will reduce the possibility of Zn atom aggregation and roughen IMC layer, and achieve the purpose of refining IMC layer [93]. Xing et al. [94] investigated the effect of Al_2_O_3_ nanoparticles on the microstructure of Sn-Zn solder. The results showed that the microstructure of Sn-Zn eutectic was more fine and uniform after adding micro nanoparticles into β-Sn matrix. Al_2_O_3_ nanoparticles can inhibit the growth of coarse dendrite Sn-Zn eutectic structure and refine the grains of composite solder. The literature [95] shows that the microstructure of Sn-Zn solder and Sn-Zn-Bi composite solder is significantly refined by Ni nanoparticle, but the IMC layer is not refined obviously.

Table 4 summarizes the effect of trace element addition on the microstructure of In-based/Sn-Bi/Sn-Zn solder. It shows that the addition of metal materials, rare earth and nanoparticles can effectively refine the matrix structure of low-temperature solder and reduce the size or quantity of Bi-rich or Zn-rich. The microstructure of solder directly affects the reliability of solder joint. The smaller the grain size of solder is, the more uniform the growth direction of interface is, and the better reliability of solder joint is. The refined grain can ensure that the internal structure of the solder joint is more compact and can effectively resist certain external pressure, which can improve the reliability of the solder joints.

**Table 4.** Effect of trace element addition on the microstructure of In-based/Sn-Bi/Sn-Zn solder.

Solder	Element	wt. %	Reflow/°C	Aging/h	thermal cycles/t	Grain Size	Bi-rich phase	Zn-rich phase	reliability	Referen-ce
In	Bi	22	85–200			↓				[50]
	Sn	48	160			↓↓			√√	[51]
Sn-Bi	P	1				↓			√	[54]
	Cu	1			2000	↑				[55,56]
	In	2.5				↓	↓			[57,63]
	Zn	1					↓			[58]
	Ni	1		150		↓				[59]
	Al	2.3				↓				[60]
	Sb	1.5				↓			√	[62]
	Cr	0.2				↓↓				[64]
	CuZnAl	0.5				↓				[66,67]
	La	0.5		1000		↓↓			√√	[68]
	Ce	0.5				↓				[69]
	Ag, Re	1, trace					↓			[71]
	Ag nanoparticles	0.4					↓		√	[72]
	Cu_6_Sn_5_ nanoparticles	trace					↓		√	[73]
	CNTs	0.03				↓↓				[74,75]
	GNSs	0.04					↓↓			[76]
	Ti nanoparticles	0.1				↓			√	[114]
Sn-Zn	Ag	1.5						↓		[77]
	Bi	3	220					↓		[78]
	Cu	1.5						↓		[79]
	Al	0.09						↓		[80]
	Bi, Cr	3, 0.5						↓↓	√√	[80,82]
	Bi, Ag	3, 0.5						↓		[84]
	Sb	0.1						↓		[86,87]
	Ti	0.05						↓		[89]
	Ce (La)	0.5						↓		[90]
	Bi, Pr	3, trace						↓		[92]
	ZrO_2_ nanoparticles	trace					↓		√	[93]
	Al_2_O_3_ nanoparticles	trace					↓			[94]
	nano Ni	trace					↓			[95]

Notes:↓ = slightly decreased ↓↓ = remarkably decreased ↑ = slightly increased ↑↑ = remarkably increased √ = slightly improved √√ = remarkably improved

## 4. Mechanical properties

### 4.1. In-based low temperature lead-free solder

In electronic components, solder joints not only play the role of electrical connection, but also play the role of mechanical support [96]. Therefore, solder joints must have excellent mechanical properties in the actual production. At present, electronic products are gradually developing towards the direction of micro element, and the size of solder joints is increasingly required. Wu et al. [97] investigated the relationship between the strength and Ag content of In-Ag solder joints. The experimental results showed that the shear strength of In-Ag solder joints increased with the increase of Ag content. In belongs to soft metal and has small shear strength. According to the fine grain strengthening Hall-Petch theory, the equation is described as follows [98]:

(5) $$\sigma =\sigma_{0}+Kd^{-\frac{1}{2}}$$

**Figure 8.**
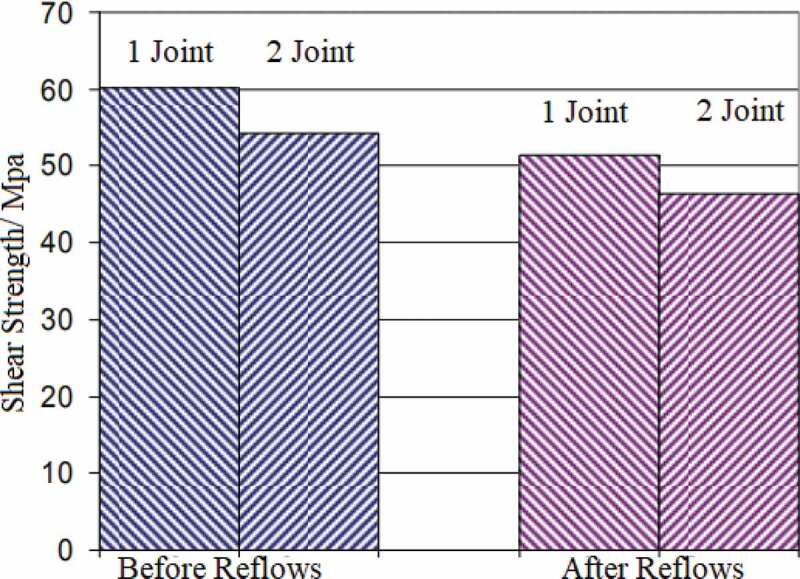
Shear strength change of solder joints after reflow.

Where σ represents the yield strength, $\sigma_{0}$ represents the yield strength of a single crystal, *K* represents a constant that depends on the material, *d* represents the size of grain. The grain size became smaller after trace amount of Ag was added to the In solder, which enhanced the strength of solder joints. Solid-state interface reactions between 50In-50Pb solder and a Cu-Fe alloy attached to an electroplated Au layer were investigated by Vianco et al. [99]. The annealing temperatures and times were 70, 100, 135, 170 °C and 1–2000 h, respectively. The results showed that the shear resistance of solder joint increased slightly after a short annealing, but the strength of solder joint began to decrease with the annealing time increased. The Au at the interface dissolves into the solder and forms (Au, Cu)In_2_ compound. The compound is implanted at the interface and grows into intermetallic compound. It has good ductility and plays a role of dispersion strengthening in the solder joint. The mechanical properties of the solder joint got improved. With the increase of annealing time, the intermetallic compound layer began to thicken and other brittle compounds were formed, which reduced the mechanical properties of the solder joints. The mechanical properties of the solder joint of alloy In and Au substrate after low temperature bonding at 200 °C were studied by Won et al. [100]. The results are shown in Figure 8. The results show that the solder joint has high shear strength (> 20MPa), and the shear strength of the solder joint does not change significantly after repeated refluxing at 206 °C. The solder joint shows high reliability. Noor et al. [15] studied the growth of intermetallic compound layer between In-Bi-Zn solder and Cu substrate. They showed that the intermetallic compound interface formed between In-Bi-Zn solder and Cu was composed of Cu_5_Zn_8_ and Cu_11_In_9_ composite layer. On the In-Bi-Zn interface of Sn/Cu substrate, Cu_5_Zn_8_ phase was better than Cu_6_Sn_5_ phase. Jin et al. [52] investigated the relationship between the mechanical properties of In-Bi low temperature solder and its composition. The results showed that the cross-sectional area of the tensile specimen with the increase of In content, showing a high plasticity. The tensile strength of In-Bi alloy decreases with the increase of In content. The decrease is probably caused by the deformation caused by recrystallization.

### 4.2. Sn-Bi low temperature lead-free solder

**Figure 9.**
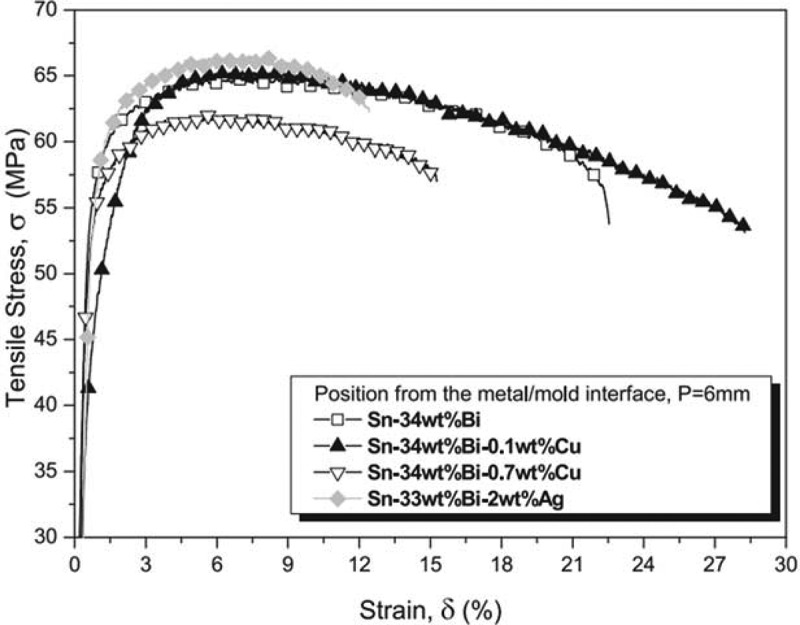
Tensile load-displacement curve of Sn-Bi-Cu solder joint. Reprinted with permission from Jun Shen, et al. [107]. Copyright 2014 Elsevier.

Zhang et al. [101] found that some Bi elements precipitated from Cu_3_Sn compound in Sn-Bi/Cu solder joints, which lead to the brittle failure of the samples during the tensile process. It was found that the mechanical properties of Sn-Bi solder joints increased first and then decreased with the increase of Ag content [102]. It was attributed to the effect of fine-grain strengthening. The Sn_3_Ag compound is formed in the filler metal when the content of Ag is less. The compound is granular or acicular. It is evenly distributed in the matrix, which can refine the structure, improve the tensile strength and play the role of fine-grain strengthening. However, Sn_3_Ag begins to segregate and grow into plate or block when the amount of Ag is more than 1 wt.%. The large size of Ag_3_Sn compound will decrease the tensile strength of the solder joint according to the hall match formula. Wang et al. [103] believed that Zn element can improve the mechanical properties of solder joints, and it is confirmed by experiments. Zn can refine the structure of solder and improve the mechanical properties. However, the Zn atoms begin to enrich when the Zn element is added too much. The Zn-rich phase is needle-like. It can reduce the mechanical properties of solder joints. Zhang et al. [27] studied the effect of Sb element on the mechanical properties of Sn-Bi solder joints. They found that the shear of solder joints was significantly improved when the amount of Sb added was more than 2.4 wt %. Sb dissolves in Sn and Bi matrix, which plays the role of solid solution strengthening and changing the structure of solder. As a result, Bi segregation and the proportion of eutectic structure produced are significantly reduced, the shear strength of solder joint is increased. Zhu et al. [104] added Cr as a reinforcing phase to Sn-Bi solder. They found that trace Cr can significantly improve the mechanical properties of solder joints, and change the fracture mode from brittle fracture to ductile fracture. The researchers attributed it to the dispersion strengthening mechanism. The CrSn_2_ intermetallic compound produced as the second phase particles dispersed in the solder matrix. It impeded the grain boundary sliding and dislocation movement and improved the shear strength of the solder joint [105]. Chen et al. [26] reported that the addition of In and Ag to Sn-Bi alloys showed good mechanical properties due to the role of Bi as a strong solid solution element in Sn matrix. The intermetallic compounds formed can be used as intensifiers and grain refiners. Gain et al. [106] added high melting point metal Ni to the low temperature Sn-Bi solder to form Sn-Bi-Ni composite solder. They found that the melting point of the composite solder increased, and the mechanical properties of the solder joint were improved. A Ni-Cu compound has formed on the surface of Cu substrate after Ni element was added in the solder. It can strengthen the equilibrium phase and refine the structure. Therefore, the mechanical properties of the solder joint will get improved. However, the excessive Ni element and Sn will form brittle compound, which will deteriorate the mechanical properties of the solder joint. Shen et al. [107] reported the research on the properties of Sn-58Bi solder joint by adding Cu element, and the experimental results are shown in Figure 9. The figure showed that the mechanical properties of Sn-Bi solder joint were improved after appropriate amount of Cu atom were added. Cu atoms can refine the structure of Sn-Bi solder. Most of the Bi-rich phase becomes a small spherical structure instead of a rod-shaped sheet. A small number of Cu_6_Sn_5_ phase with peculiar shape is wrapped in the Bi-rich phase, which can promote the refinement of Bi crystal branch and improve the tensile strength of the solder joint. The mechanical properties of Sn-Bi solder joints were studied by Roh et al. [108]. The results showed that the shear strength of the solder joints increased with the increase of reflow time. Xu et al. [109] studied the mechanical properties of Sn-Bi/Sn-3.0Ag-0.5Cu/Cu solder joint after long time aging. They found that the hardness of Sn-Bi/Sn-3.0Ag-0.5Cu/Cu solder joint was less than that of Sn Bi/Cu solder joint after aging. The addition of Sn-3.0Ag-0.5Cu system made Sn-Bi/Cu solder joint fracture become brittle after refluxing.

It has been reported that rare earth elements can improve the mechanical properties of solder [110]. It is found that the mechanical properties of Sn-Bi solder joints can be effectively improved by adding rare earth elements [111]. RE refine the grains in the structure, and lead to the improvement of alloy properties. However, the shear force of the solder joint decreased after aging treatment, but the shear force is still higher than that of Sn-Bi alloy solder without adding rare earth elements.

The influence of nano particles on the mechanical properties of Sn-Bi solder joints is reported in the literatures [112,113]. The results show that the mechanical properties of Sn-Bi/Cu solder joints can be significantly improved by adding micro amount SiC nanoparticles. The SiC nanoparticles can prevent the dislocation from sliding, refine the solder grain at the same time. It can reduce the probability of crack source, and enhance the plasticity of solder. The influences of doping Ti nanoparticles on microstructure and properties of Sn-58Bi solder was studied by Jiang et al. [114]. The result shows that the shear force of Sn-58Bi-0.1Ti solder is higher than that of Sn-58Bi after multiple reflows.

**Figure 10.**
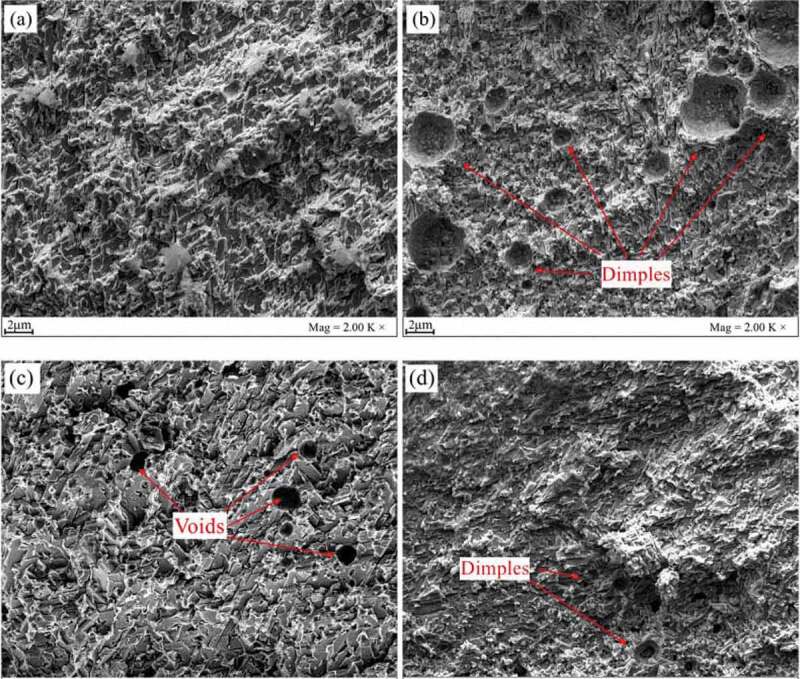
Tensile fracture morphologies of Sn-58Bi-*x*Mo (*x* = 0, 0.25) solder joints after 0 and 1200 thermal cycles: (a) Sn-58Bi, 0; (b) Sn-8Bi-0.25Mo, 0; (c) Sn-58Bi, 1200; (d) Sn-58Bi-0.25Mo, 1200. Reprinted with permission from Li Yang, et al. [118]. Copyright 2019 Elsevier.

The literature [115] shows that CNTs and GNSs can connect two Bi-rich phase grains or directly embed in Bi-rich phase. The CNTs and GNSs can play a good role in pinning and buffering. The fracture due to stress concentration will be decreased, and the mechanical properties of solder joints will be improved. The effect of CNTs coated with Ni on the properties of Sn-Bi solder was reported by Yang et al. [116]. They pointed out that CNTs and Ni_3_Sn_4_ compound strengthen the composite material together, which makes the tensile strength of solder joint enhanced. The tensile strength and elongation decrease with the further increase of the content of Ni-CNTs due to the existence of CNTs clusters and the increase of brittleness. Lee et al. [117] investigated the mechanical properties of Sn-58Bi solder joints by multi walled carbon nanotubes (MWCNTs) coated with Ag. They found that the addition of trace Ag-MWCNTs nanoparticles improved the shear strength of Sn-58Bi solder joints. The Ag-MWCNT can refine the Sn-58Bi solder crystal and play a structural role in the solder joint, thus the shear strength of the solder joint was improved. Li et al. [118] studied the effect of nano-Mo particles on the mechanical properties of Sn-Bi solder joints after aging. Figure 10 shows the fracture surface of the experimental sample. It can be seen from the figure that the fracture mode of Sn-Bi-Mo solder joint is the mixed fracture of toughness and brittleness. There are many dimples inside the solder joints. The ductile fracture characteristics completely disappear after the thermal cycle. The shear force and tensile strength of Sn-Bi and Sn-Bi-Mo solder joints are decreased with the increase of the number of thermal cycles, but the mechanical properties of Sn Bi-Mo solder joints are still better than that of Sn-Bi solder joints. The microstructure of solder joints becomes rough and IMC layer is thicker after multiple thermal cycles. A large number of Kirkendall voids are produced.

### 4.3. Sn-Zn low temperature lead-free solder

The mechanical properties of eutectic Sn-9Zn solder and the properties of hypoeutectic Sn-4Zn and hypereutectic Sn-12Zn solder have been studied by Garcia et al. [119]. The results show that the microstructure of eutectic solder is mainly spherical-like Zn-rich, while the microstructure of hypoeutectic and hypereutectic is mainly needle-like Zn-rich, which is the main reason why the mechanical properties of Sn-9Zn are higher than that of Sn-4Zn and Sn-12Zn. Daly et al. [120] pointed out that the mechanical properties of Sn-Zn solder were improved after appropriate amount of Ag were added. The density of solid-phase crystal nucleus and the free arrangement of crystal nucleus increased during the crystallization process of Sn-9Zn solder. It hinders the formation of huge needle-like structure. The micro Ag-Zn compound has the function of dispersion strengthening, which improves the mechanical properties of solder joints. Kim et al. [121] found that Bi dissolved and diffused in Sn-rich phase with the increase of Bi content, the shear strength of Sn-Zn solder joint increased, but the plasticity of solder joint decreased. Yu et al. [122] found that the mechanical properties of Sn-Zn lead-free solder will be improved with the addition of Al, and the melting of Sn-Zn solder can be reduced. Al element has the effect of solid solution strengthening in Sn-Zn solder, which improves the tensile strength of Sn-Zn alloy solder joint. Das et al. [123] studied the effect of Cu on the mechanical properties of Sn-Zn solder joints. The results showed that Cu atoms can refine the structure and form uniform and dense intermetallic compounds. It can ensure the mechanical properties and good air tightness of solder joints. Wang et al. [124] studied the effect of micro In element on the mechanical properties of Sn-Zn solder joints. The results showed that the shear force of Sn-Zn solder joints was reduced after micro in element were added. Hu et al. [125] investigated the growth of IMC between solder and Cu matrix after 0.1 wt.% Cr were added into Sn-9Zn Lead-free solder alloy and the effect of isothermal aging on IMC. They found that the composition of IMC was the same as that of Sn-9Zn/Cu during the soldering process, but Cr inhibited the growth of IMC during the aging process. The precipitated Zn-Cr phase decreased the diffusion rate of Zn atom. It can produce more uniform IMC, and enhance the shear strength of the solder joint. Luo et al. [126] also studied the effect of Cr element on the mechanical properties of Sn-Zn solder joint. The addition of Al element can improve the wettability and mechanical properties of Sn-Zn composite solder [127]. On this basis, some researchers added Cu and Ni elements to synthesize the quaternary composite solder and study the relationship between the mechanical properties and the content of the fourth element. The results show that the mechanical properties of the composite solder can be effectively improved by adding appropriate amount of Cu or Ni elements. Cu (Ni) is easy to react with Zn atom to form Cu-Zn (Ni-Zn) intermetallic compound, which can refine or even disappear the coarse needle-like Zn-rich phase and refine the structure to a certain extent. According to the theory of fine grain strengthening, the mechanical properties of Sn-Zn-Al-Cu (Ni) composite solder are improved. The effect of Sb on the mechanical properties of Sn-Zn-Al composite solder was studied by Basaty et al. [128]. The results showed that the tensile strength of Sn-Zn-Al-Sb solder joint increased with the addition of proper Sb element due to the effect of Sb solution strengthening and dispersion strengthening of fine-grained IMC, so the ultimate tensile strength got increased. It is reported that Ge element can effectively improve the oxidation resistance of Sn-Zn-Bi composite solder, reduce the resistivity of the solder, and increase the mechanical properties of Sn-Zn-Bi solder joints as a whole [129]. Sharif et al. [130] studied the effect of substrate on the solder joint strength of Sn-Zn-Bi solder. The researcher plated a layer of Ni on the surface of Cu substrate by chemical plating. He found that the solder joint strength of Sn-8Zn-3Bi on Ni-Cu substrate was stronger than that of Sn-8Zn-3Bi on Cu substrate. Ni-Zn compound layer produced is relatively uniform. They can reduce the formation factors of crack source and improve its mechanical properties. Liu et al. [131] reported the effect of Ni element on Sn-Zn-Bi composite solder. The Ni element reacts with Zn atom to form Ni_5_Zn_2_ compound layer, which inhibits the diffusion of Zn atom to the substrate. It can inhibit the growth of compound layer. Also, it will make IMC layer uniform and improve the mechanical properties of solder joints. Sharif et al. [132] thought that the spalling of intermetallic compound layer between Sn-Zn-Bi-Ag alloy solder/Au/Ni (Au-Zn) is controlled by Ag content. The researcher found that the formation of brittle Ni-Zn compound is reduced when the Ag content is 0.3 wt%. The ductile brittle transition mode of solder joint fracture is delayed, and the mechanical properties of solder joint were improved. The interface reaction of eutectic Sn-9Zn solder on Cu substrate electroplated with Au/Ni during aging was studied by Yoon et al. [133]. It was found that a layer of AuZn_3_ intermetallic compound was formed during aging. The IMC layer gradually peels off with the increase of aging time, and a layer of Ni_5_Zn_21_ IMC layer is produced. The compound layer grows up gradually, which makes the shear strength of the solder joint decrease gradually. In order to prevent the decline of the mechanical properties of the solder joint, the peeling off of AuZn_3_ compound layer should be avoided as far as possible to ensure the reliability of Sn-9Zn/Au/Ni/Cu solder joint of the solder joint.

**Figure 11.**
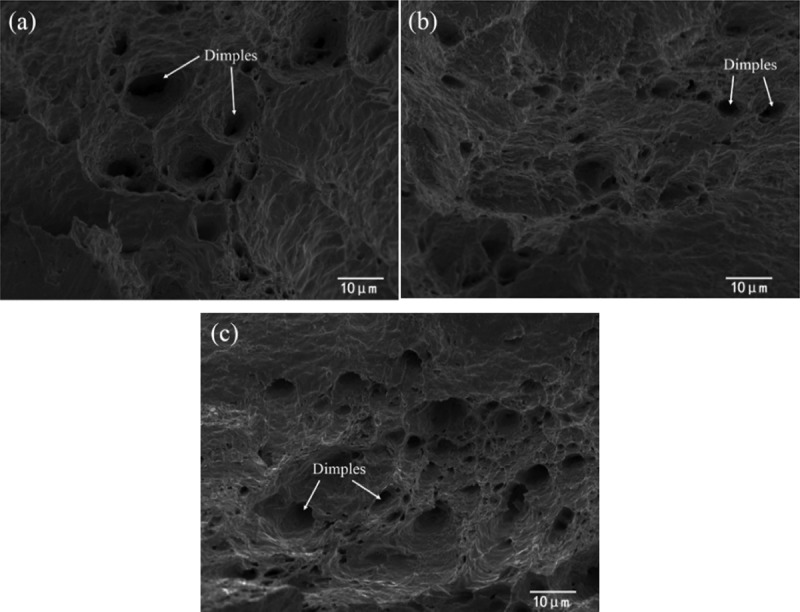
SEM fracture surfaces of Sn-Zn solders doped with Al_2_O_3_ nanoparticles: (a) Sn-9Zn, (b) Sn-9Zn-0.5Al_2_O_3_, (c) Sn-9Zn-1Al_2_O_3._

The oxidation activity of rare earth elements (La, Pr, Ce, etc.) is very high. The addition of trace RE elements can refine the structure of β – Sn phase rod-like Zn-rich phase. It is the main reason for rare earth elements to improve the mechanical properties of solder [134]. According to the Sn-Yb phase diagram [135], the composition point of Yb and Sn is close to the reaction zone on the side of Sn after trace Yb elements were added to Sn Zn solder. The YbSn_3_ compound phase formed is a stable phase. The fine particles gathered at the grain boundary and played an obvious strengthening role. Hu et al. [136] studied the relationship between the amount of Nd added and the mechanical properties of Sn-Zn solder joint. It can be found that many dense and uniform ‘fluffy’ fine eutectic structures are produced inside the solder joint when the amount of Nd added is 0.1 wt.%, which makes the tensile shear strength increase. As shown in Figure 11(c), the mechanical properties of solder joints decrease because of the enrichment of Nd element in IMC, which weakens the modification of Nd element. The relationship between Pr and the mechanical properties of Sn-Zn solder joints was studied by Zhao et al. [137]. The results showed that appropriate Pr could make the acicular IMC(Cu_5_Zn_8_) layer more uniform, improve the air tightness of the joint surface and improve the mechanical properties.

Shen et al. studied the effect of nanomaterial addition on the mechanical properties of Sn-Zn solder joints [93]. The results showed that the mechanical properties of solder joints could be significantly improved when appropriate ZrO_2_ nanoparticles was added. ZrO_2_ nanoparticles can be embedded in the solder matrix and play a role in pinning the grain boundary due to the small size of nanoparticles. It can prevent the occurrence of dislocation to a certain extent so as to improve the shear strength of the solder joint when the solder joint is subject to external force. The influence of Al_2_O_3_ nanoparticles on the mechanical properties of Sn-Zn solder joints was reported in the literature [138]. The results are shown in Figure 11. It can be seen from the SEM image that the fracture morphology of all solders shows the existence of tough dimples. The size of the dimples is smaller and the distribution is more uniform when the amount of Al_2_O_3_ nanoparticles is 1 -wt. %. It was pointed out that the tensile strength of the solder joint increased by about 10.9% compared with that of the common Sn-Zn solder joint when Al_2_O_3_ nanoparticles were added. The researchers attributed it to the use of Al_2_O_3_ nanoparticles, which made the pore size smaller and distributed uniformly. Therefore, the mechanical properties of solder joints got improved. It was found that the shear strength of Sn-9Zn solder joint increased after Ni nanoparticles were added. Nano Ni particles can refine the microstructure of solder and reduce the internal crack source of solder joint. The fracture mode of solder joint is mainly ductile fracture [139].

Table 5 summarizes the effect of trace element addition on the mechanical properties of In-based/Sn-Bi/Sn-Zn solder. It can be found from Table 5 that the addition of metal materials, rare earth and nanoparticles can effectively increase the mechanical properties of solder joints, which directly ensure the better reliability of solder joints. However, the mechanical properties of solder joints decrease due to more brittle Cu_6_Sn_5_ compounds were produced at the interface after aging or reflowing, thus the reliability of solder joints got worse.

**Table 5.** Effect of trace element addition on the mechanical properties of In-based/Sn-Bi/Sn-Zn solder.

Solder	substrate	Element	Wt.%	Reflow/°C	Shear strength	Tensile force	reliability	Reference
	Au				↓			[100]
In	Cu	Bi, Zn	32.7, 0.5		↑		√	[52]
Sn-Bi	Cu	Bi	30–40		↑↑			[101]
	Cu	Bi	58	200		↓↓		[101]
	Cu	Ag	6.7		↑ (20.6%)		√	[102]
	Cu	Zn	2		↑			[103]
	Cu	Sb	2.4		↑ (20.1%)			[27]
	Cu	Cr	0.2			↑ (10%)		[104]
	Cu	In	4		↓ (5%)			[26]
	Cu	Zn, Cu	2, 0.1			↑↑ (22.3%)		[107]
	Cu	Bi	58	80		↓ (10%)		[109]
	Cu	SiC Nanoparticles	trace		↑		√	[112]
	Cu	Ti Nanoparticles	0.1		↑ (6.9%)			[114]
	Cu	GNSs	0.1			↑ (15%)		[115]
	Cu	Ni-CNTs	0.05		↑ (6.7%)			[116]
	Cu	Ag-MWCNTs	0.1		↑ (16.3%)			[117]
	Cu	Nano-Mo	0.25		↑↑ (37.9%)		√√	[118]
	Cu	Zn	9		↑↑ (33.3%)			[119]
Sn-Zn	Cu	Ag	1.5			↑↑ (49.1%)		[120]
	Cu	Al	0.5		↑ (20%)			[123]
	Cu	In	1		↑			[124]
	Cu	Cr	0.1		↑		√	[125,126]
	Cu	Al, Sb	0.5, 1.5		↑↑ (73,3%)			[128]
	Ni-Cu	Bi	3		↑ (18%)			[130]
	Cu	Nd	0.06			↑		[135]
	Cu	Pr	0.1		↑↑ (75%)			[137]
	Ni/Au	ZrO_2_ nanopartic-les	1		↑ (2%)			[93]
	Cu	Al_2_O_3_ nanopartic-les	1			↑ (4.8%)		[138]
	Au/Cu	nano Ni	trace		↑		√	[139]

Notes:↓ = slightly decreased ↓↓ = remarkably decreased ↑ = slightly increased ↑↑ = remarkably increased √ = slightly improved √√ = remarkably improved

## 5. Oxidation resistance

### 5.1. In-based low temperature lead-free solder

In is an active element, but it does not react with oxygen under normal conditions. The literature shows that there is little research on the oxidation resistance of In solder.

### 5.2. Sn-Bi low temperature lead-free solder

Sn-Bi solder will not react with oxygen at room temperature, but oxidation may occur during the soldering. Little is known about the oxidation resistance of Sn-Bi solder. The effects of high-temperature aging on a novel hybrid bonding layer consisted of Cu nanoparticles and a eutectic Bi-Sn solder powder was studied by Usui et al. [140]. The results show a relatively high amount of oxides in the bonding layer. Some cracks appeared in the bonding layer after aging at 225 °C for 100 h. The cracks became the channel of oxidation reaction, which produce more oxides and decreased the reliability of solder joint. The effect of Al/P on the oxide film thickness of Sn-Bi-Zn (SBZ) solder was investigated by Wang et al. [141]. The oxide on the surface of SBZ is the product of high concentration of oxygen and Zn. Al^3+^ accumulated on the surface of liquid solder and forms oxide film after Al were added, which prevent the further oxidation of SBZ. Teng et al. [142] found that the oxidation resistance of Sn-Bi-Ag solder is the best when the content of Ge is 0.007 wt. %.

### 5.3. Sn-Zn low temperature lead-free solder

**Figure 12.**
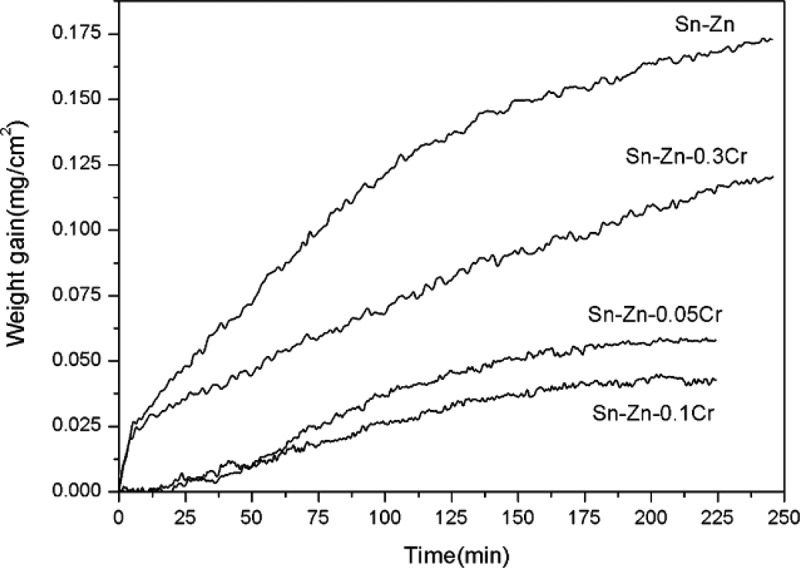
Thermogravimetric analysis of Sn-9Zn-*x*Cr alloys (*x* = 0, 0.05, 0.1, 0.3). Reprinted with permission from Xi Chen, et al. [144]. Copyright 2008 Elsevier.

Zn element easily reacts with O_2_, which makes the oxidation resistance of solder containing Zn very poor (Table 6). It is a unique property of solder containing Zn [143]. The oxide film formed by the reaction of Zn and oxygen will hinder the flow of the molten Sn-Zn solder and reduce the filling ability of the liquid solder. Therefore, improving the oxidation resistance of Sn-Zn solder is one of the main methods to improve the performance of the solder [43]. Chen et al. [144] found that Sn-Zn solder has the best oxidation resistance when Cr content is 0.1 wt.%, and the experimental results are shown in Figure 12. It can be seen from the figure that the growth rate of oxidation film is the slowest when the Cr content is 0.1 wt %. It indicates that Zn segregates in the surface of all specimens. Cr segregates in the subsurface layer, which results in thinner oxidation film. Also, Chen et al. [145]reported that the oxidation resistance is enhanced with the addition of 0.5% Ga. Chang et al. [146] studied the relationship between the oxidation resistance of Sn-9Zn solders and the content of Ag and In. They found that both Sn-9Zn-0.5Ag and Sn-9Zn-0.5Ag-1In solders have a higher oxidation resistance than that of Sn-9Zn solder. The relationship between the oxide thickness and the element content can be expressed as an equation:

(6) $$\begin{array}{rl} & Y=1.453+0.265X^{2}\;for\;9Zn-Sn\\ & Y=0.031+0.157X-0.020X^{2}\;for\;5.0Ag-9Zn-Sn\\ & Y=-0.083+0.283X-0.015X^{2}\;for\;1\;In-0.5Ag-9Zn-Sn \end{array}$$

**Table 6.** Partial pressure of common solder oxides.

	Bi_2_O_3_	In_2_O_3_	SnO_2_	PbO	ZnO	CuO
298 K	9.0 × 10^−39^	5.2 × 10^−39^	6.9 × 10^−92^	7.0 × 10^−67^	2.3 × 10^−112^	4.7 × 10^−46^
400 K	1.4 × 10^−41^	4.4 × 10^−50^	6.5 × 10^−66^	2.2 × 10^−47^	2.3 × 10^−82^	6.5 × 10^−32^
500 K	1.5 × 10^−31^	2.1 × 10^−38^	8.9 × 10^−57^	5.9 × 10^−38^	4.6 × 10^−63^	6.8 × 10^−24^
600 K	8.6 × 10^−35^	1.4 × 10^−35^	1.5 × 10^−40^	1.8 × 10^−28^	5.4 × 10^−51^	2.0 × 10^−18^

Where Y represents weight gain ratio, X represents aging time. It can be seen from the above equation that with the increase of aging time, the growth rate of oxide film thickness is lower than that of Sn-9Zn solder when appropriate amount of Ag and In elements are added. The effect of Bi on the oxidation resistance of Sn-Zn solder was reported by Jiang et al. [147]. They point out that the oxidation resistance becomes poorer when 1 wt.% Bi was added into the solder. Bi can form solid solution in Sn matrix, which makes Sn harden and crack easily. It has high distortion energy, which promotes Zn diffusion. Zhou et al. [148] studied the effect of trace rare earth element Nd on the oxidation resistance of Sn-8Zn-3Bi solder. The results showed that the oxidation resistance of the composite solder was the best when the content of Nd was 0.1 wt%. A layer of oxide film (Nd_2_O_3_) is formed by the rapid reaction of Nd element with oxygen. The oxide film is wrapped on the surface of the molten composite solder, which blocks the entry of oxygen. It reduced the contact between Zn and oxygen, and improved the oxidation resistance of the solder.

Table 7 summarizes the effect of trace element addition on the oxidation resistance of In-based/Sn-Bi/Sn-Zn solder. It can be seen from Table 7 that the oxidation resistance of Sn-Zn solder can be improved by adding metal materials, rare earth, etc., which can improve the reliability of solder joint. Poor oxidation resistance is a special property of Zn containing solder, which is also an important reason to limit its use. The addition of some elements can effectively prevent the oxidation of Zn in solder joints, thus the reliability of solder joint can get improved.

**Table 7.** Effect of trace element addition on the oxidation resistance of In-based/Sn-Bi/Sn-Zn solder.

Solder	Element	wt. %	Oxidation resistance	reliability	Reference
			↓		[140]
Sn-Bi	Al	0.005	↑		[141]
	Ge	0.007	↑	√	[142]
Sn-Zn	Cr	0.1	↑		[144]
	Ga	0.5	↑↑		[145]
	Ag	0.5	↑		[146]
	Ag, In	0.5, 1	↑↑	√√	[146]
	Bi	1	↑		[147]
	Nd	0.1	↑		[148]

Notes:↓ = slightly decreased ↓↓ = remarkably decreased ↑ = slightly increased ↑↑ = remarkably increased √ = slightly improved √√ = remarkably improved

## 6. Conclusions

The research status of low temperature lead-free solder is comprehensively evaluated. The alloy elements (Cu, Zn, Bi etc.), rare earth elements (Ce, La, Pr etc.), nanoparticle (CNTs, GNSs etc.) are summarized. It is found that some alloy elements, rare earth elements and nano materials can refine the internal structure of solder. It can reduce the size of intermetallic compound particles and reduce the thickness of interface to some extent. The addition of reinforcement elements can improve one or the whole performance of low temperature solder, but the opposite view has been put forward, which is mainly related to the way and content of reinforcement phase. At present, there are very few reports on low-temperature In-based solders, especially on the solders containing nanomaterials and rare earth elements.
